# Testosterone and Cardiovascular Health: Physiology, Pharmacokinetics and Clinical Implications

**Published:** 2026-04-02

**Authors:** Gaithrri Shanmuganathan, Nora Lyang, Sandeep Gill, Devendra K. Agrawal

**Affiliations:** 1 Department of Translational Research, College of the Ostepathic Medicine of the Pacific, Western University of Health Sciences, Pomona, CA, USA; 2 Department of Internal Medicine, Loma Linda University Health, Loma Linda, CA, USA; 3 Southwest Healthcare Medical Education Consortium, Temecula Valley Hospital, Temecula, CA, USA

**Keywords:** Arrhythmia, Atherosclerosis, Atrial Fibrillation (AFib), Cardiovascular Disease (CVD), Dihydrotestosterone (DHT), Erythrocytosis, Follicle Stimulating Hormone (FSH), Formulation, Heart Failure, Inflammation, Luteinizing Hormone (LH), Plaque Burden, Testosterone, Testosterone Replacement Therapy (TRT)

## Abstract

Cardiovascular disease (CVD) is becoming increasingly prevalent in the United States, prompting an interest in biological and hormonal contributors to risk. Although testosterone has long been implicated in cardiovascular risk, the nature of this relationship is complex and is not fully understood. Furthermore, the use of testosterone replacement therapy has increased over the years, especially in male hypogonadism. Like endogenous testosterone, the influence of testosterone replacement therapy on CVD risk remains unclear as findings from multiple studies have been inconsistent. Some studies proposed that testosterone replacement therapy-induced erythrocytosis may contribute to an increased risk of CVD, however, this association remains unconfirmed. Building on this proposed mechanism, some studies have found certain testosterone replacement therapy formulations to have increased rates of erythrocytosis while other studies found similar effects across all formulations. In this review, we outline the physiology of endogenous testosterone, its role in cardiovascular health and the current evidence surrounding testosterone replacement therapy and its relationship with CVD. We will then review the different testosterone replacement therapy formulations and evaluate their potential influence on CVD risk.

## 1. Introduction

Cardiovascular disease (CVD) is defined as the disease of blood vessels and the heart. Some major examples of CVD include atrial fibrillation (AF), coronary heart disease (CHD) and heart failure (HF) [1]. According to the American Heart Association (AHA), the overall prevalence of CVD is expected to rise from 11.3% in 2019 to 15% by 2050 [1]. While clinical CVD is expected to affect 45 million adults, CVD risk factors such as hypertension is projected to affect 184 million (more than 61%) adults by 2050 [1]. A research study by Faridi et al. demonstrated an increased 10-year predictive risk of CVD or existing CVD in 3 out of 10 U.S. adults between ages 30 and 79, with more than 90% of those in ages 65 years and up [2]. In younger middle-aged adults, there is an increased 30-year CVD risk in those without any existing CVD. Of these numbers, the highest risk demographic tends to be amongst men, and those who are of Hispanic or Black descent [2]. On a global scale, the prevalence of CVD was observed to increase from 271 million cases in 1990 to 523 million cases in 2019 [3]. In terms of mortality rate stemming from heart disease in the last 50 years, there has been an 81% decline in age-adjusted mortality from all ischemic heart disease, and an 89% decline in age-adjusted mortality from acute myocardial ischemia [4]. However, as of 2022, there has been an 81% increase in age-adjusted mortality from other heart disease subtypes including hypertensive heart disease, heart failure (HF), and arrhythmia [4].

Given these rising trends, it is crucial to understand the role of hormonal and biological risk factors in the development of CVD. Emerging evidence suggests dysregulation of testosterone levels may contribute to the development of CVD [5,6]. Testosterone influences cardiovascular physiology through various interacting pathways, which could help explain the variation in study findings [7]. Additionally, there has been an increasing interest in the use of testosterone replacement therapy (TRT) particularly for the treatment of male hypogonadism [8]. Due to this interest, multiple studies have been done to determine the cardiovascular safety of TRT use but findings have been inconsistent, leading to its ongoing debate [9]. To provide a cohesive understanding of the effect of testosterone on cardiovascular health, this review will examine endogenous testosterone physiology, formulation pharmacokinetics and current cardiovascular outcome data.

## 2. Endogenous Testosterone Biology

### 2.1 Physiology

Testosterone is the primary male androgen and is synthesized in the Leydig cells under luteinizing hormone (LH) stimulation [10,11]. Its production begins when circulating cholesterol is transported into the mitochondria of Leydig cells by the steroidogenic acute regulatory protein (StAR) [12]. Inside the mitochondrial matrix, cholesterol side-chain cleavage enzyme (CYP11A1) converts cholesterol into pregnenolone. As pregnenolone moves into the smooth endoplasmic reticulum, it is converted to progesterone by 3β-hydroxysteroid dehydrogenase (3HSD3B), and then to androstenedione by 17α-hydroxylase/17,20-lyase (CYP17A1) [12]. Androstenedione is subsequently converted into testosterone by 17β-hydroxysteroid dehydrogenase type 3 (HSD17B3) [12].

Once formed, testosterone can follow different metabolic pathways. A substantial portion is converted to the more potent androgen dihydrotestosterone (DHT) by 5α-reductase in peripheral tissues such as the liver, skin, adrenal, kidney, prostate and genital skin [13]. Testosterone can also be aromatized to estradiol in tissues such as adipose tissue, liver, brain, bone, breasts and testes [13,14].

**Figure 1:**
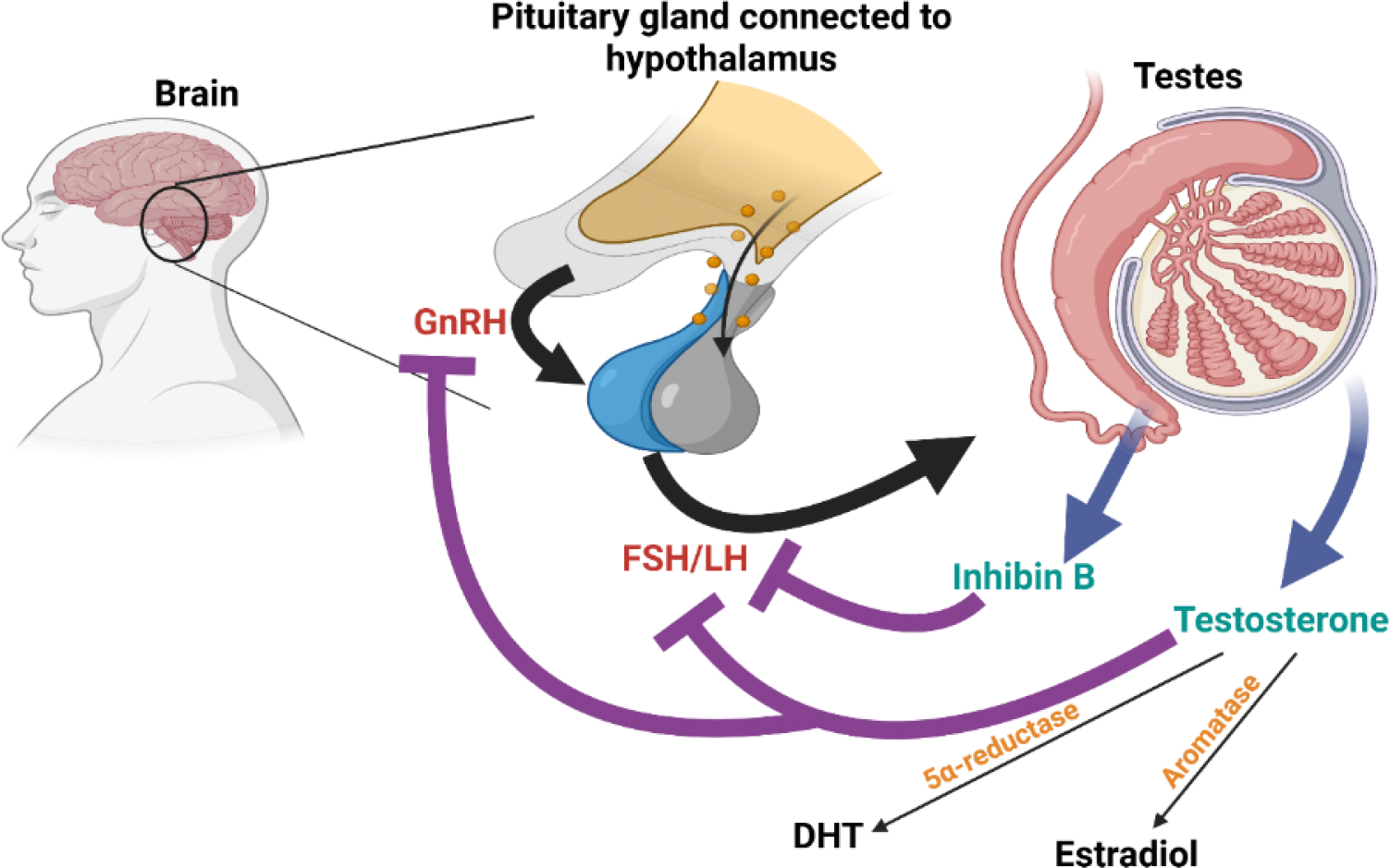
Pulsatile Gonadotropin-releasing hormone (GnRH) release from hypothalamus stimulates release of follicle-stimulating hormone (FSH) and luteinizing hormone (LH) from anterior pituitary gland. LH activates release of testosterone from Leydig cells in testes, whereas FSH stimulates Sertoli cells to promote spermatogenesis and the release of inhibin B. Circulating testosterone can be aromatized to estradiol or converted to dihydrotestosterone (DHT) by the action of 5-alpha reductase. GnRH and LH secretion is inhibited by bioavailable testosterone, DHT and estradiol, while inhibin B selectively blocks FSH. These coordinated actions allow for maintenance of homeostasis. Created in BioRender.

In circulation, about 30–44% of testosterone is tightly bound to sex hormone-binding globulin (SHBG), while 54–68% is loosely bound to albumin [15,16]. The remaining testosterone exists in an unbound form, also known as free testosterone, which comprises 0.5–3% [16]. Bioavailable testosterone includes the albumin-bound and free fractions, which can enter target tissues and activate androgen receptors (AR) [17]. SHBG-bound testosterone does not readily dissociate and is therefore not considered bioavailable. Total testosterone reflects all three forms [17]. Testosterone that is not converted to other hormones or bound to a receptor is metabolized in the liver to inactive metabolites such as androsterone and dehydroepiandrosterone and is ultimately excreted through the kidneys or the gut [14] (Figure 1).

Testosterone production is tightly regulated by the hypothalamic-pituitary-gonadal (HPG) axis to maintain hormonal balance, and disruptions at any point in this axis can contribute to hypogonadism [18]. Gonadotropin-releasing hormone (GnRH) is secreted in a pulsatile manner from the hypothalamus into the hypophyseal portal system, stimulating the anterior pituitary gland to release LH and follicle-stimulating hormone (FSH) [19]. These gonadotropins are also released in a pulsatile manner, although FSH pulsatility is less pronounced. LH acts on Leydig cells to promote testosterone synthesis, while FSH stimulates Sertoli cells of the testes to support spermatogenesis [19,20].

Once testosterone levels reach an optimal level, bioactive testosterone including albumin-bound and free testosterone, along with DHT, binds to AR in the hypothalamus and pituitary gland to suppress further GnRH and LH secretion. FSH suppression occurs through inhibin B produced by Sertoli cells as well as estradiol feedback [19, 21]. SHBG-bound testosterone and inactive metabolites do not participate in this feedback loop [22].

Beyond hormonal regulation, testosterone secretion follows a circadian rhythm driven by both central and peripheral mechanisms. Levels are higher in the morning, and lowest in the evening. Centrally, testosterone interacts with ARs in the suprachiasmatic nucleus (SCN), and peripherally, Leydig cells have their own intrinsic clock that contributes to this rhythm. Factors such as poor sleep, aging, obstructive sleep apnea (OSA) and obesity can disrupt this synchrony and impair testosterone production [23–25]. Recognizing this diurnal pattern is important for accurate diagnosis and management.

### 2.2 Epidemiologic Trends

In males, testosterone production shows a characteristic early-life pattern marked by a brief hormonal surge known as minipuberty. This occurs around 1–3 months of age and continues until about 6 months [26,27]. During this period, total testosterone levels can reach concentrations like those seen during puberty (172 ± 78 ng/dL), whereas free testosterone remains comparatively low (7.7 ± 4 pg/mL or 0.45 ± 0.20%) [26]. This temporary increase in total testosterone plays a role in early testicular development and helps prime the reproductive endocrine system for later function [26]. After this phase, testosterone levels fall back to prepubertal concentrations, typically below 1 nmol/L [28]. With the onset of puberty, generally between ages 9 and 14, both free and total testosterone begin to rise sharply. Levels increase by nearly seven-fold before reaching adult concentrations of around 15 nmol/L by ages 16–17, at which point they stabilize [28, 29].

Although testosterone is the primary androgen in males, it is also present in females at much lower concentrations. In a cross-sectional study of individuals aged 6–20 years, Senefeld et al. [29] reported that total testosterone increased from 1.9 ng/dL to 516 ng/dL in males, whereas the increase in females was much smaller, from 2.4 ng/dL to 29.5 ng/dL [29]. Males reached their plateau around age 17, while females reached theirs earlier, at about age 14 [29]. These differences highlight testosterone’s more prominent developmental role in males.

Testosterone levels begin to decline gradually in both sexes during early adulthood [30]. In men, this decline usually occurs later during the third and fourth decades of life, whereas in women it tends to occur earlier, between the second and third decades [31].

A longitudinal study by Banica et al. [30] found annual decreases in total testosterone, free testosterone and DHT of 0.85%, 1.31% and 1.03%, respectively, accompanied by a 0.62% yearly rise in SHBG [30]. The age-related increase in SHBG contributes to a steeper decline in free testosterone than in total testosterone. Findings from the Baltimore Longitudinal Study of Aging similarly showed age-related increases in SHBG in both men and women. In women, SHBG followed a U-shaped trajectory, with a slight decline before menopause and a sharper increase afterwards. In men, SHBG increased steadily, with a faster increase after age 70 [32].

Bioavailable testosterone, as measured by ammonium sulfate precipitation (mBT), decreased consistently with age in both sexes, with a more pronounced decline in men. Total testosterone decreased before menopause in females and then remained stable or slightly increased afterward. In males, total testosterone generally remained stable until around age 70, after which it declined more rapidly [32]. These hormonal changes in aging men have been associated with alterations in metabolic and vascular risk profiles, although causality has not been established [32].

### 2.3 Diagnosing Male Hypogonadism

According to the American Urological Association (AUA), Endocrine Society and U.S. Food and Drug Administration (U.S. FDA), one of the primary indications for TRT is confirmed hypogonadism due to organic causes, including primary or secondary hypogonadism stemming from disorders of the hypothalamus, pituitary gland or testes [33–35].

The diagnosis of hypogonadism follows a stepwise approach. It begins with obtaining a morning (7AM to 11AM), preferably fasting, total testosterone level<300 ng/dL on two separate days, along with the presence of signs or symptoms consistent with hypogonadism, such as fatigue, depression, anemia, gynecomastia and decreased libido [33,34]. Patients should then be evaluated for reversible secondary causes of low testosterone, including medication use such as glucocorticoids and opioids, obstructive sleep apnea (OSA), obesity, and systemic illness. After these factors are addressed, total testosterone should be measured again on two separate occasions. If levels remain <300 ng/dL, further evaluation is warranted [33,34].

In patients with conditions that affect SHBG including obesity, type 2 diabetes mellitus, aging, HIV, liver disease, thyroid disorders or exogenous androgen use, or those with borderline total testosterone (200–400 ng/dL), free testosterone level should be measured using equilibrium dialysis or calculated using a validated formula incorporating total testosterone, SHBG, and albumin [34].

**Figure 2:**
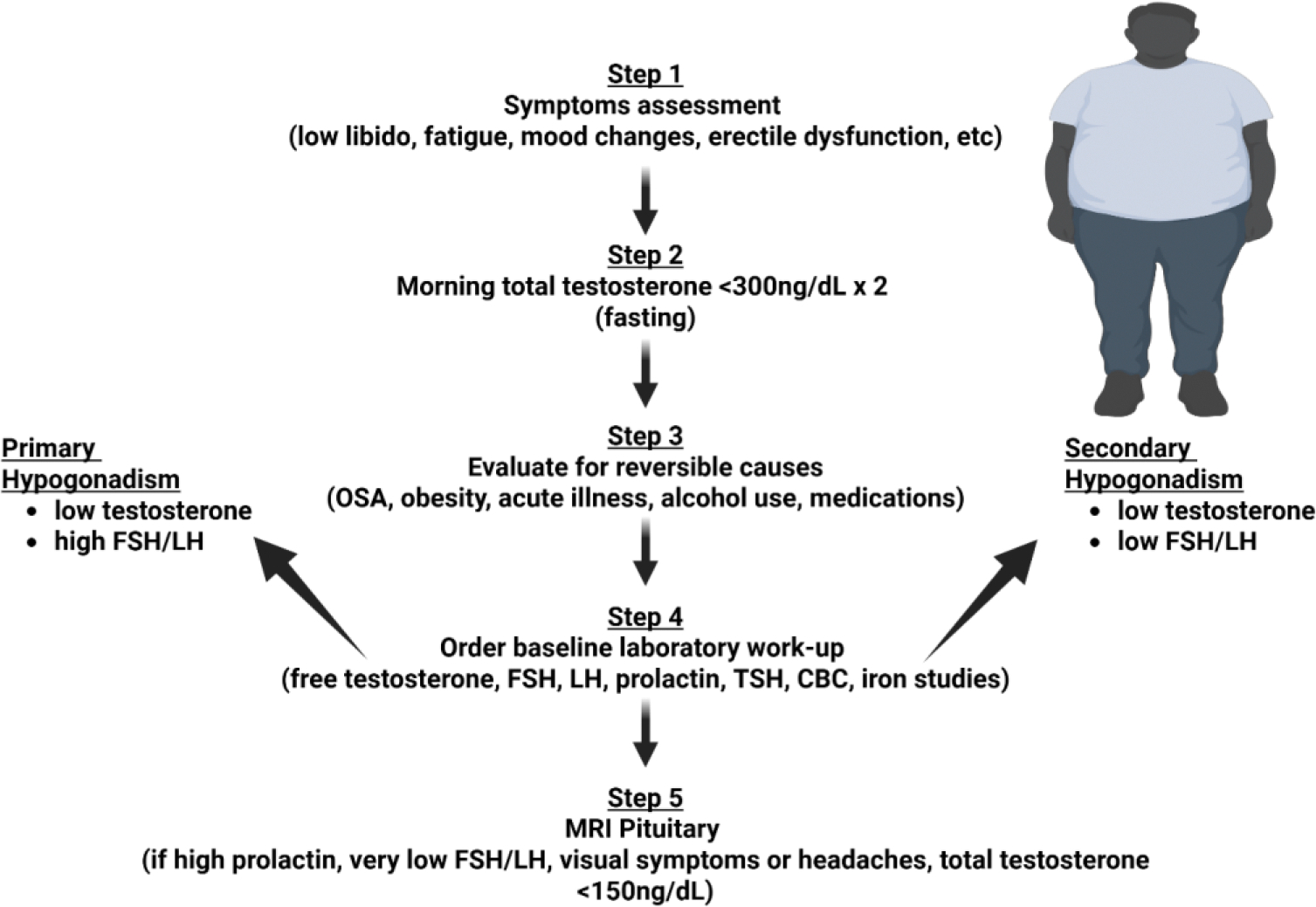
Evaluation begins with symptom assessment followed by two fasting total testosterone measurements on separate days if symptoms are suspicious of hypogonadism. Secondary and reversible causes are identified and addressed. If suspicion for organic causes of hypogonadism persists, further labs are ordered including free testosterone, FSH, LH, prolactin, CBC and iron studies to evaluate for primary or secondary causes of hypogonadism. Brain MRI is recommended if patient presents with headaches, vision changes, inappropriately low or normal FSH/LH or markedly high prolactin. Created in BioRender.

Following this, LH and FSH are obtained to differentiate between primary hypogonadism resulting from impaired testicular function, and secondary hypogonadism resulting from pituitary or hypothalamic causes. If LH is low or inappropriately normal, prolactin should be checked to evaluate for pituitary pathology. Brain MRI is recommended when there are symptoms suggesting mass effect including diplopia or headaches, or if there are lab abnormalities involving prolactin or other pituitary hormones. Additional labs may include iron studies to check for iron overload, and assessment of other pituitary hormones such as thyroid stimulating hormone (TSH) and adrenocorticotropic hormone (ACTH) [34] (Figure 2).

In summary, both biochemical evidence of low testosterone and compatible clinical symptoms must be present to justify treatment with TRT [34]. This ensures therapy is limited to patients with true primary or secondary hypogonadism, rather than functional-related or age-related testosterone decline [35].

## 3. Cardiovascular Actions of Testosterone: Mechanistic Overview

### 3.1 Vascular and Endothelial Effects

Testosterone affects vascular physiology through several mechanisms. In the genomic pathway, testosterone enters the cell and binds to nuclear AR, resulting in gene transcription and activation of PI3K/Akt pathway. This, in turn, stimulates phosphorylation of endothelial nitric oxide synthase (eNOS), and increases nitric oxide (NO) production, promoting vasodilation [36,37].

Testosterone also acts through nongenomic pathways. When it binds ARs on the plasma membrane, it facilitates the formation of a complex with c-Src and caveolin-1, which again activates PI3K/Akt and enhances eNOS activity and NO production [37]. In the microvasculature, testosterone can also promote peroxynitrite formation, triggering cyclic GMP (cGMP) signaling and contributing further to vasodilation [38].

Beyond its effects on endothelial signaling, testosterone directly influences vascular smooth muscle. It can inhibit L-type calcium channels and activate potassium channels, lowering intracellular calcium concentrations and resulting in vascular smooth muscle relaxation [39,40]. A similar ion channel effect occurs in cardiac myocytes, where testosterone activates ultra-rapid potassium channel, accelerating repolarization and shortening QT interval, thereby linking testosterone to cardiac electrophysiologic changes [41].

### 3.2 Hematologic Effects

From a hematologic standpoint, testosterone stimulates erythropoiesis through genomic mechanisms. It binds to early hematopoietic cells in the bone marrow, promoting development of myeloid progenitor lines [42]. This increases erythropoietin (EPO) production, which then drives red blood cell (RBC) formation. To support this RBC formation, testosterone enhances iron availability by suppressing hepcidin and increasing ferroportin-mediated iron release and utilization [42–44].

### 3.3 Lipid metabolism

Testosterone also influences lipid metabolism through genomic and non-genomic mechanisms. Through its genomic effects, testosterone upregulates the activity of hepatic lipase, which increases hydrolysis of phospholipids within high-density lipoprotein cholesterol (HDL-C) [45,46]. It also alters low-density lipoprotein cholesterol (LDL-C) composition by shifting particles toward smaller and denser forms. Collectively, these actions can lead to reductions in LDL-C, HDL-C and total cholesterol levels [47].

Testosterone also promotes activation of liver X receptor alpha (LXRα), which upregulates ATP-binding cassette transporter A1 (ABCA1) and apolipoprotein E (APOE) [48]. These proteins allow for potential anti-atherogenic effects by inducing cholesterol efflux from macrophages and reducing foam cell formation [48,49]. In addition to its genomic effects, testosterone can alter lipid handling through non-genomic mechanisms by increasing hepatic lipid oxidation and enhanced secretion of very-low-density lipoprotein triglyceride (VLDL-TG) [50].

### 3.4 Inflammatory Effects

Testosterone can have both pro-inflammatory and anti-inflammatory effects, and the direction of its action depends on the type of cell involved and circulating hormone level.

Through genomic pathways, testosterone can suppress transcription factor such as nuclear factor kappa B (NF-κB) and may modulate AP-1 (cJun). Clinical studies also demonstrated that TRT may reduce expression in pro-inflammatory mediators including tumor necrosis factor alpha (TNF-α) and interleukin-1 beta (IL-1β), while increasing anti-inflammatory interleukin-10 (IL-10) production [51–53].

Through non-genomic mechanisms, testosterone binding can activate G-protein-coupled receptor (GPCR) and phospholipase C, triggering a rapid calcium influx [54]. Depending on the specific cell type, this rapid influx may either activate or inhibit the extracellular signal-regulated kinase (ERK)1/2 signaling, an important component of the mitogen-activated protein kinase (MAPK) pathway [55].

## 4. Testosterone Formulations, Pharmacokinetics and Cardiovascular Implications

Several routes of TRT formulations are available, including transdermal patches, gels, intranasal gel, oral capsules, intramuscular and subcutaneous injections. Understanding the pharmacokinetics of each formulation is important when choosing the most appropriate option for a patient [34,56].

### 4.1 Injectable Formulations

Three injectable testosterone formulations are currently approved by the U.S. FDA, including testosterone enanthate, testosterone undecanoate, and testosterone cypionate. All are administered intramuscularly, most commonly into the gluteal region [57–59].

Dosing varies depending on the indication, but for male hypogonadism, testosterone cypionate and enanthate are commonly given at 50–400 mg every 2 to 4 weeks [34]. Testosterone undecanoate follows a longer dosing schedule, starting with 750mg at baseline, again at 4 weeks, and then every 10 weeks thereafter [59].

Testosterone cypionate and enanthate share similar pharmacokinetics. Both are esterified in oil, which allows for slow absorption. These short-acting formulations have a half-life of roughly 8 days, with slightly more variability observed in the testosterone enanthate [57,58]. Testosterone cypionate generally reaches peak serum levels between days 2 and 5, and levels decline to a trough by days 13–14 [56,60]. The large difference between the peak right after an injection and the low point before the next dose often creates a noticeable “roller coaster” effect, which some patients describe as shifts in mood or libido [61].

Testosterone undecanoate is also esterified in oil but is considered long-acting given its longer half-life of about 34 days [62,63]. Peak serum levels generally occur around day 7, though the reported range is broad, anywhere between 4 to 42 days [59]. Levels then decline gradually, and steady state is typically achieved after the third injection. All three injection formulations undergo hepatic metabolism with most metabolites excreted in the urine and the remainder eliminated through the feces [59].

### 4.2 Transdermal, Oral, Intranasal and Buccal Formulations

Topical testosterone gels are available in various strengths, including 1%, 1.62% and 2% formulations [64–66]. They are applied once daily to areas such as the shoulder, upper arms, abdomen, or thighs depending on the specific product. Typical starting doses are 50 mg for the 1% gel, 40.5 mg for the 1.62% gel, and 40 mg for the 2% gel, with adjustments made based on serum testosterone levels [64–66].

Transdermal patches are another commonly used option and are usually started at 2–4 mg daily, applied to non-scrotal skin [67]. Gels reach peak concentrations within 2 to 4 hours after application while patches provide a more continuous 24-hour delivery [68,70]. After patch removal, testosterone exhibits a terminal half-life of approximately 1.3 hours [70]. Both gels and patches maintain minimal trough [68,69]. Similar to other formulations, they are metabolized in the liver and excreted mainly in the urine [70].

Oral testosterone provides an alternative route with distinct absorption characteristics compared with transdermal products. Two oral testosterone formulations are FDA-approved for male hypogonadism, including testosterone undecanoate and methyltestosterone [71–73]. Testosterone undecanoate is typically started at 112.5 mg twice daily although this varies by brand [73]. It should be taken with meals to facilitate lymphatic absorption [73]. The undecanoate ester has an elimination half-life of about 2.5 hours, while the active testosterone formed has a half-life of 10 to 110 minutes [70,73]. Peak concentrations occur around 5 hours, with serum testosterone levels declining over the 12-hour dosing interval, reaching lower levels before the next dose [73]. Methyltestosterone is started 10–50 mg daily, although higher doses (up to 400 mg) may be needed due to significant first-pass hepatic metabolism [71,72]. Peak serum concentrations are achieved rapidly after ingestion [71,72].

A rapid-acting option is intranasal testosterone, which differs from oral and transdermal agents in both dosing frequency and pharmacokinetics. Intranasal testosterone gel is dosed at 11 mg three times daily [74]. It is absorbed quickly, with peak levels reached roughly 40 minutes after administration and trough levels by 6–8 hours [74]. Its half-life is 10 to 110 minutes, similar to other formulations [70]. It is metabolized to DHT and estradiol through typical androgen pathways, with DHT increasing proportionally during treatment [74].

Buccal testosterone is another short-acting route and is administered at a starting dose of 30 mg twice daily [75, 76]. Maximum serum concentrations are reached 10–12 hours after placement, and levels fall below the normal range within 2 to 4 hours after removal [75]. Steady state is usually achieved after the second day of twice-daily dose, and the elimination half-life is 1.75 hours [75, 77].

### 4.3 Pellets Formulation

The only FDA-approved subcutaneous pellet is the crystalline testosterone pellet. Typical dosing ranges from 150–450 mg (usually 2–6 pellets) implanted every 3 to 6 months [78]. Testosterone is released slowly, with the greatest amount delivered during the first month, followed by a gradual taper over the next several months. Levels remain therapeutic for approximately 4–6 months, and the elimination half-life is 70–75 days [79]. A brief rise in serum testosterone occurs within the first 12 hours after placement before transitioning into a sustained-released phase [78].

### 4.4 Formulation-Specific Cardiovascular Risks

TRT has been the subject of long-standing debate regarding its cardiovascular safety, largely because available studies have produced conflicting results.

The most recent and comprehensive evidence comes from the Therapy for Assessment of long-term Vascular Events and Efficacy ResponSE in Hypogonadal Men (TRAVERSE) study which used the 1.62% testosterone gel [80]. In this randomized trial, TRT users experienced higher rates of atrial fibrillation (AFib) and nonfatal arrhythmias compared with placebo, but the therapy was still considered noninferior for major adverse cardiac events (MACE), suggesting no excess risk of myocardial infarction, stroke, or cardiovascular death [80].

A more recent retrospective cohort study by Connelly et al. [81] challenged this conclusion. With a longer follow-up period designed to reflect real-world TRT exposure, the study reported a 54% increase in unadjusted MACE risk (hazard ratio 1.54; 95% confidence interval 1.18–2.00) and a 55% increase in adjusted MACE risk (hazard ratio 1.55; 95% confidence interval 1.19–2.01) [81]. Unlike the TRAVERSE trial, this study included a range of formulations, such as oral, transdermal, and intramuscular testosterone. Although cardiovascular risk appeared higher with both transdermal and injectable formulations, statistical significance was reached only for the transdermal group [81].

Differential effects of specific formulations were also noted in an earlier observational study by Layton et al. [82], which evaluated 544,115 TRT users. Compared to gels, injectable testosterone was associated with higher risks of cardiovascular events (hazard ratio 1.26), hospitalization (hazard ratio 1.16) and death (hazard ratio 1.34), while patches did not meaningfully differ from gels [82]. However, the study had several limitations, including possible unmeasured confounding, variable adherence among formulations, and the lack of confirmation that patients met diagnostic criteria for hypogonadism. In addition, there was no untreated control group for comparison [82].

While cardiovascular risk remains unresolved, the relationship between TRT and erythrocytosis is more consistent. Several studies have shown that TRT can raise hematocrit, though the degree of risk differs across formulations [82]. Both Scala et al. [84] and Rivero et al. [83] demonstrated increases in hematocrit, but Rivero et al. [83] further showed that this effect was specific to intramuscular injections and did not occur with intranasal gels, suggesting that large fluctuations in serum testosterone might contribute to erythropoiesis [83, 84].

It is still uncertain whether TRT-related erythrocytosis directly increases cardiovascular risk. However, two observational studies found that men who developed polycythemia while receiving TRT had significantly higher rates of MACE compared with men who did not develop polycythemia, raising concern that elevated hematocrit may be part of the mechanism connecting TRT with adverse cardiovascular outcome [85,86].

## 5. Effects of Testosterone on Clinical CVD Phenotypes

### 5.1 Atherosclerotic Disease

#### 5.1.1 Endogenous Testosterone and Lipid Profile

Several observational studies have examined how endogenous testosterone relates to different lipid fractions. In a retrospective cohort study of Chinese men with a mean age of 56 years, Zhang et al. [87] demonstrated that higher total testosterone levels were associated with lower triglycerides, total cholesterol and LDL-C, after adjusting for confounding factors including age, BMI, blood pressure, glucose, and thyroid function [87]. Conversely, HDL-C showed the opposite pattern, with higher testosterone levels associated with higher HDL-C in the adjusted analysis [87].

Makinen et al. [88] reported similar findings in their study of 1619 men aged 40 to 69 years. Testosterone showed a positive correlation with HDL-C (r = 0.24, p <0.0001) and inverse relationship with total cholesterol (r = −0.06, p <0.03), triglycerides (r = −0.30, p <0.0001) and body mass index (r = −0.34, p <0.0001) [88]. No meaningful association was seen with LDL-C (r = 0.05, p = 0.09) [88].

Harris et al. [89] further demonstrated that an increase in HDL-C was accompanied by higher total testosterone, while higher total cholesterol and triglycerides were associated with lower testosterone levels [89]. Their multivariate analysis also identified positive associations between testosterone and both HDL-C and LDL-C [89]. Taken together, these studies show that despite some variations across lipid fractions, low endogenous testosterone is associated with a more atherogenic lipid pattern.

#### 5.1.2 Testosterone Replacement Therapy (TRT) and Lipid Profile

Several studies have also examined how TRT influences lipid metabolism, with findings showing considerable variation across different lipid fractions.

In a case control study of Japanese men with hypogonadism, Kato et al. [90] reported that compared to the untreated group, men treated with TRT had significant reductions in triglycerides at 1-, 3-, and 5-years post-treatment. TRT in this cohort was also significantly associated with improvements in glycemic parameters, including lower fasting blood glucose at 3- and 5-years post-treatment, and significantly lower hemoglobin A1c at the 1-, 3-, and 5-years post-treatment [90]. Notably, this study did not identify meaningful changes in total cholesterol or HDL-C [90].

Other studies have reported different patterns. In a non-randomized retrospective cohort study, Clift et al. [91] observed decreases in HDL-C, triglycerides, and triglyceride-to-HDL ratio after 1 year of TRT [91]. Similarly, a double-blind placebo-controlled trial across 12 academic centers in the United States reported a significant reduction in HDL-C after 1 year of TRT compared to placebo [47]. This trial also found significant reductions in LDL-C and total cholesterol, while triglyceride levels remained unchanged.

Collectively, these studies show that TRT can influence lipid fractions in different ways depending on population characteristics, study design and formulation used. Although many of these changes reach statistical significance, the absolute changes were small and unlikely to be clinically meaningful. Therefore, TRT should not be used solely to treat dyslipidemia.

#### 5.1.3 Endogenous Testosterone and Plaque Burden

To better understand how endogenous testosterone may influence atherosclerosis progression, it is important to examine its relationship with coronary and carotid plaque burden, both of which play key roles in overall cardiovascular risk.

Khazai et al. [92] evaluated the relationship between circulating sex hormones and carotid intima-media thickness (cIMT) in a large multi-ethnic cohort study of men without known CVD [92]. Their analysis showed that both bioavailable testosterone and total testosterone were higher in men with lower cIMT measurements [92]. In contrast, using data from the Tromsø study, Vikan et al. [93] reported a modest inverse association between total testosterone and total carotid plaque area after adjusting for age, systolic blood pressure, smoker status, and use of lipid-lowering drugs (p = 0.033) [93]. They did not find an association between free testosterone and plaque area, and SHBG showed only a borderline inverse trend (p = 0.054) [93]. In their prospective analysis, baseline sex hormone levels did not predict changes in plaque area or cIMT, which the authors attributed partly to increasing use of lipid-lowering agents [93].

Similarly, Srinath et al. [94] showed that low testosterone levels were associated with several cardiometabolic risk factors including higher body mass index, diabetes, lower HDL-C, hypertension, and greater waist circumference (p = 0.01) [94]. However, testosterone levels were not related to mean cIMT in either adjusted or unadjusted analyses [94].

Studies assessing coronary plaque burden have shown mixed findings. Khazai et al. [92] also found an inverse relationship between free testosterone and the likelihood of having a coronary artery calcium score (CACS) greater than zero, and between total testosterone level and log-transformed CACS [92].

In contrast, Trumble et al. [95] studying a physically active Tsimane population with minimal metabolic disease, found that higher testosterone levels were associated with higher CACS values. In their analysis, men with a CACS greater than 100 had approximately 20% higher testosterone levels than those with lower CACS (p = 0.007) [95]. The authors suggested that inverse associations reported in industrialized populations may reflect confounding effect from obesity and other metabolic conditions [95].

Overall, these findings illustrate the inconsistent relationship between endogenous testosterone and both coronary and carotid plaque burden. Because this association appears to be influenced by underlying comorbidities and cardiovascular risk factors, endogenous testosterone is best viewed as a risk marker rather than a causal driver of atherosclerosis progression.

#### 5.1.4 Testosterone Replacement Therapy and Plaque Burden

Clinical trials have also evaluated how TRT affects plaque burden. One controlled trial examined 1 year of testosterone gel in hypogonadal men aged 65 years and older. At the 12-month follow-up, men receiving TRT had a significantly greater increase in noncalcified coronary artery plaque volume (p = 0.003) and total plaque volume (p = 0.006) compared with the placebo group, based on coronary CT angiography [96]. In contrast, a randomized clinical trial by Basaria et al. [97] assessed the impact of 3 years of TRT on progression of subclinical atherosclerosis in men with low to low-normal testosterone levels. Their study found no relationship between TRT and the rate of change in cIMT or CACS [97].

Overall, these trials show mixed results. Some evidence suggests TRT may promote increases in plaque burden, whereas other studies have not demonstrated measurable effects on atherosclerosis progression. Additional research is needed to clarify whether these findings reflect clinically meaningful cardiovascular risk.

### 5.2 Heart Failure

#### 5.2.1 Endogenous Testosterone and Heart Failure

Several studies have explored how endogenous testosterone relates to HF. Across most of these studies, low endogenous testosterone has been linked to worse HF outcomes or higher HF risk.

In a prospective observational cohort study, Yoshihisa et al. [98] stratified men into quartiles based on testosterone: > 631 ng/dl, 462 - 631 ng/dl, 300 – 462 ng/dl, and ≤ 300 ng/dl [98]. Men in the lowest quartile had significantly lower peak VO2, higher mortality, lower exercise capacity and more myocardial damage. Supporting these findings, Zhan et al. [99] reported a cross-sectional study that higher serum testosterone levels were associated with decreased HF risk (p < 0.001), particularly in men older than 50 years of age. This association persisted after adjusting for relevant confounders (p = 0.01) [99]. Similar results were observed in the Cardiovascular Health Study, where lower total testosterone was associated with increased HF risk (hazard ratio 1.14; 95% CI, 1.01–1.28) [100].

However, not all studies show the same pattern. In the UK Biobank analysis, testosterone levels were not associated with incident HF, although SHBG demonstrated an unexpected inverse association with incident HF [101]. Therefore, while most studies suggest an inverse association between endogenous testosterone and HF risk or functional status, these findings represent associations rather than causal relationships.

#### 5.2.2 Testosterone Replacement Therapy and Heart Failure

There are relatively few studies evaluating how TRT affects HF, and although some data suggests possible benefits, the overall evidence remains limited.

Regarding safety, Cannarella et al. [102] performed a meta-analysis examining TRT use in hypogonadal men with advanced HF (NYHA class II - III) or coronary artery disease. TRT use was associated with increased oxygen consumption, but there were no significant changes in blood pressure, NYHA class, left ventricular ejection fraction (LVEF), rehospitalization rates, mortality, or quality of life [102]. These findings suggest that TRT can be used safely in certain HF populations, though additional studies are needed.

The question of whether TRT improves HF outcomes remains unresolved. A meta-analysis of randomized controlled trials by Tao et al. [103] evaluated the effects of chronic TRT on exercise capacity and cardiac function in patients with chronic HF. Overall, TRT did not significantly affect quality of life, cardiac function, exercise capacity or major clinical outcomes [103]. Similarly, a randomized controlled trial by Navarro-Peñalver et al. [104] in men with heart failure with reduced ejection fraction (HFrEF) showed no significant effects in LVEF, N-terminal-pro-B-type natriuretic peptide (NT-proBNP) levels, exercise tolerance, or HF symptoms [104].

On the other hand, a more recent systematic review reported that some individual trials demonstrated improvements in metabolic measures including fat mass, lean muscle mass, as well as improved muscle strength and aerobic performance with TRT [105]. However, these findings were limited by short follow-up periods and small sample sizes. In summary, TRT appears to be reasonably safe in HF populations, but current evidence does not support a clear, consistent benefit on cardiac function or clinical outcomes.

### 5.3 Arrhythmias

#### 5.3.1 Endogenous Testosterone and Arrhythmias

Recent studies have examined whether endogenous testosterone plays a role in arrhythmia risk, particularly atrial fibrillation (AFib). Using data from the Atherosclerosis Risk in Communities (ARIC) study, Berger et al. [106] observed that men in the highest quartile of total testosterone initially appeared to have a slightly lower risk of AFib compared with those in the lowest quartile, although this finding was not statistically significant [106]. The association reversed, and higher testosterone levels were associated with an increased AFib risk adjusting for a broader set of clinical factors including body mass index, HF, coronary artery disease, kidney function, diabetes, antihypertensive use and systolic blood pressure (hazard ratio 1.33, 95% CI 1.07, 1.66) [106].

A post-hoc analysis from the ASPirin in Reducing Events in the Elderly (ASPREE) trial further supported this association. Tran et al. [107] reported that men with higher levels of testosterone had a significantly higher risk of incident AFib, even after excluding participants who developed major adverse cardiovascular events (MACE) or HF [107]. Men who developed AFib during follow-up also had higher baseline testosterone compared with those who remained free of AFib. Similarly, findings from the Multi-Ethnic Study of Atherosclerosis (MESA) study showed that men with higher bioavailable testosterone had a higher incidence of AFib (hazard ratio = 1.32, 95%CI = 1.01, 1.74; p = 0.044 when comparing the highest to the lowest tertile) [108].

However, not all studies align with this pattern. An analysis of the Framingham Heart Study reported that lower testosterone levels were associated with a higher risk of AFib in older men. For participants aged 55–69 years, each 1-standard deviation (SD) decrease in testosterone was linked to a higher risk of incident AFib (hazard ratio 1.30, 95% CI 1.07–1.59), and this association was even stronger in men aged 80 years and older (hazard ratio 3.53, 95% CI 1.96–6.37) [109]. Taken together, these studies suggest that the relationship between testosterone and AFib may not be linear. A potential U-shaped association is possible, with both low and high levels potentially conferring risk [109]. Age, comorbidities, and other cardiovascular risk factors likely modify this relationship.

Data on other types of arrhythmias remains limited. In a UK Biobank analysis, Xu et al. [110] found that higher calculated free testosterone and higher SHBG levels were associated with an increased risk of ventricular arrhythmias (hazard ratio 1.18, 95% CI 1.01–1.37, and hazard ratio 1.27, 95% CI 1.07–1.52, respectively). Elevated SHBG levels were also associated with a higher risk of bradyarrhythmia (hazard ratio 1.17, 95% CI 1.05–1.29) [110]. These results should be interpreted cautiously, as the study relied on a single hormone measurement and may be subject to potential confounders. Overall, the evidence linking endogenous testosterone with arrhythmia risk is mixed, and further studies with repeated hormone measurements and longitudinal assessment are needed to better define these associations.

#### 5.3.2 Testosterone Replacement Therapy and Arrhythmias

Multiple studies have evaluated whether TRT influences the risk of atrial arrhythmias, but evidence remains inconsistent. In the TRAVERSE trial, which evaluated cardiovascular outcomes in hypogonadal men with elevated cardiovascular risk, TRT was found to be noninferior to placebo for MACE [80]. However, AF occurred more frequently in the TRT arm than in the placebo arm (3.5% vs 2.4%, p = 0.02) [80]. Nonfatal arrhythmias requiring intervention were also common among TRT users (5.2% vs 3.3%, p = 0.001) [80].

To further assess arrhythmia risk, Greenberg et al. [111] conducted a retrospective cohort study to using eligibility criteria like the TRAVERSE study. Their analysis did not show a significant association between TRT-users and the risk of new-onset AF (risk ratio 1.48, 95% CI 0.93–2.37) [111]. In contrast, Blackwell et al. [112], using the TriNetX database, reported a small but statistically significantly reduction in AF risk among TRT users compared to nonusers (3.6% vs 4.0%; risk ratio 0.900; p < 0.001) [112]. More recently, Bonnet et al. [113] compared cisgender hypogonadal men treated with TRT to transgender men and found that cis men receiving TRT had a higher incidence of AF over a five-year period (hazard ratio 1.27; 95% CI 1.22–1.32; p < .0001) [113].

In summary, findings vary across studies, with some suggesting an increased risk of AF and others showing no association or even reduced risk. Given these conflicting results, larger, long-term studies are needed before firm recommendations can be made regarding AF risk and TRT use.

## 6. Conclusion

The relationship between testosterone and cardiovascular disease remains complex and continues to evolve. While endogenous testosterone levels have been associated with various cardiovascular risk markers, study findings are inconsistent and do not demonstrate a causal relationship. Evidence regarding the cardiovascular safety of TRT is similarly mixed. Although several recent trials have shown no increase in MACE among appropriately selected men with hypogonadism, longer-term observational studies have reported conflicting results. A consistent finding across multiple studies is the development of erythrocytosis with TRT. It also remains uncertain whether the degree of erythrocytosis varies by formulation. Emerging data suggest that TRT-induced rise in hematocrit may contribute to downstream cardiovascular risk in certain patients, although this relationship has not been established. Given these uncertainties, TRT should be reserved for patients with well-documented hypogonadism, and clinicians should closely monitor hematocrit and cardiovascular status throughout treatment. Ultimately, longer-term, formulation-specific studies are needed to more clearly define the safety profile of TRT and guide individualized treatment decisions.

## References

[1] Joynt Maddox KE, Elkind MSV, Aparicio HJ, Forecasting the Burden of Cardiovascular Disease and Stroke in the United States Through 2050—Prevalence of Risk Factors and Disease: A Presidential Advisory from the American Heart Association. Circulation 150 (2024). 10.1161/CIR.0000000000001256 38832505

[2] Faridi KF, Malik D, Essa M, 10-Year and 30-Year Risks of Cardiovascular Disease in the U.S. Population. Journal of the American College of Cardiology [Internet] 85 (2025): 2239–49. 40499978 10.1016/j.jacc.2025.03.546

[3] Roth GA, Mensah GA, Johnson CO, Global Burden of Cardiovascular Diseases and Risk Factors, 1990–2019: Update from the GBD 2019 Study. Journal of the American College of Cardiology 76 (2020): 2982–3021. 33309175 10.1016/j.jacc.2020.11.010 PMC7755038

[4] King SJ, Wangdak Yuthok TY, Bacong AM, Heart Disease Mortality in the United States, 1970 to 2022. Journal of the American Heart Association 14 (2025). 10.1161/JAHA.124.038644 PMC12449979 40557798

[5] Mehta LS, Velarde GP, Lewey J, Cardiovascular disease risk factors in women: The impact of race and ethnicity: A scientific statement from the american heart association. Circulation [Internet] 147(2023). 10.1161/CIR.0000000000001139 PMC11196122 37035919

[6] Yeap BB, Marriott RJ, Dwivedi G, Associations of Testosterone and Related Hormones with All-Cause and Cardiovascular Mortality and Incident Cardiovascular Disease in Men. Annals of internal medicine 177(2024). 10.7326/ANNALS-25-00440 40523296

[7] Lorigo M, Mariana M, Oliveira N, Vascular Pathways of Testosterone: Clinical Implications. Journal of Cardiovascular Translational Research 13 (2019): 55–72. 31820333 10.1007/s12265-019-09939-5

[8] Muncey W, Omil-Lima D, Jesse E, Assessment of public interest and current trends in testosterone replacement therapy. International Journal of Impotence Research 18 (2021). 10.1038/s41443-021-00445-4 34007065

[9] Morgentaler A, Miner MM, Caliber M, Testosterone Therapy and Cardiovascular Risk: Advances and Controversies. Mayo Clinic Proceedings [Internet] 90 (2015): 224–51. 25636998 10.1016/j.mayocp.2014.10.011

[10] Nassar GN, Leslie SW. Physiology, testosterone [Internet]. PubMed. Treasure Island (FL): StatPearls Publishing (2023). Available from: https://www.ncbi.nlm.nih.gov/books/NBK526128/

[11] Elzenaty RN, du Toit T, Flück CE. Basics of androgen synthesis and action. Best Practice & Research Clinical Endocrinology & Metabolism 36 (2022): 101665. 35595638 10.1016/j.beem.2022.101665

[12] Zirkin BR, Papadopoulos V. Leydig cells: Formation, function, and regulation. Biology of Reproduction [Internet] 99 (2018): 101–11. 29566165 10.1093/biolre/ioy059 PMC6044347

[13] Sherbet DP, Auchus RJ. Peripheral Testosterone Metabolism. Humana Press eBooks (2007): 181–8.

[14] Handelsman DJ. Androgen Physiology, Pharmacology, Use and Misuse [Internet]. Nih.gov. MDText.com, Inc (2020). Available from: https://www.ncbi.nlm.nih.gov/sites/books/NBK279000/

[15] Fleseriu M, Hashim IA, Karavitaki N, Hormonal Replacement in Hypopituitarism in Adults: An Endocrine Society Clinical Practice Guideline. The Journal of Clinical Endocrinology & Metabolism 101 (2016): 3888–921. 27736313 10.1210/jc.2016-2118

[16] Basaria S Male hypogonadism. The Lancet 383 (2014): 1250–63. 10.1016/S0140-6736(13)61126-5 24119423

[17] Belchetz PE, Barth JH, Kaufman JM. Biochemical endocrinology of the hypogonadal male. Annals of Clinical Biochemistry 47 (2010): 503–15. 20956400 10.1258/acb.2010.010150

[18] Huijben Manou, Lock MTWT, de MO Vincent, Clomiphene citrate for men with hypogonadism: a systematic review and meta-analysis. Andrology 10 (2022): 451–69. 34933414 10.1111/andr.13146

[19] Marques P, Skorupskaite K, George JT, Physiology of GNRH and Gonadotropin Secretion [Internet]. www.ncbi.nlm.nih.gov. MDText.com, Inc.; (2018). Available from: https://www.ncbi.nlm.nih.gov/sites/books/NBK279070/

[20] O’Donnell L, Stanton P, De Kretser D. Endocrinology of the Male Reproductive System and Spermatogenesis [Internet]. Nih.gov. MDText.com, Inc. (2017). Available from: https://www.ncbi.nlm.nih.gov/books/NBK279031/

[21] Hayes FJ, Seminara SB, Decruz S, Aromatase inhibition in the human male reveals a hypothalamic site of estrogen feedback. The Journal of Clinical Endocrinology and Metabolism [Internet] 85 (2009): 3027–35. 10.1210/jcem.85.9.6795 10999781

[22] Goldman AL, Bhasin S, Wu FCW, A Reappraisal of Testosterone’s Binding in Circulation: Physiological and Clinical Implications. Endocrine Reviews 38 (2017): 302–24. 28673039 10.1210/er.2017-00025 PMC6287254

[23] Novaes LF, Flores JM, Benfante N, Analysis of diurnal variation in serum testosterone levels in men with symptoms of testosterone deficiency. The Journal of Sexual Medicine [Internet] 21 (2024): 408–13 38481019 10.1093/jsxmed/qdae026 PMC12371534

[24] Ning G, Li BN, Wu H, Regulation of testosterone synthesis by circadian clock genes and its research progress in male diseases. Asian Journal of Andrology [Internet] 27 (2025). 10.4103/aja20258 PMC12422579 40101130

[25] Wittert G The relationship between sleep disorders and testosterone in men. Asian Journal of Andrology [Internet] 16 (2014): 262. 24435056 10.4103/1008-682X.122586 PMC3955336

[26] Contreras M, Manish Raisingani, Chandler D, Salivary Testosterone during the Minipuberty of Infancy. Hormone Research in Paediatrics 87 (2017): 111–5. 28073108 10.1159/000454862

[27] Rohayem J, Alexander EC, Heger S, Mini-Puberty, Physiological and Disordered: Consequences, and Potential for Therapeutic Replacement. Endocrine reviews 45 (2024). 10.1210/endrev/bnae003 PMC11244267 38436980

[28] Khairullah A, Cousino Klein L, Ingle SM, Testosterone Trajectories and Reference Ranges in a Large Longitudinal Sample of Male Adolescents. PLoS ONE [Internet] 9 (2014). 10.1371/journal.pone.0108838 PMC4182562 25268961

[29] Senefeld JW, Lambelet Coleman D, Johnson PW, Divergence in Timing and Magnitude of Testosterone Levels Between Male and Female Youths. JAMA 324 (2020): 99. 32633795 10.1001/jama.2020.5655 PMC7341166

[30] Banica T, Verroken C, Reyns T, Early Decline of Androgen Levels in Healthy Adult Men: An Effect of Aging Per Se? A Prospective Cohort Study. The Journal of Clinical Endocrinology & Metabolism [Internet] 106 (2020): e1074–83. 10.1210/clinem/dgaa915 33382411

[31] Qaseem A, Horwitch CA, Vijan S, Testosterone Treatment in Adult Men With Age-Related Low Testosterone: A Clinical Guideline From the American College of Physicians. Annals of Internal Medicine 172 (2020): 126. 31905405 10.7326/M19-0882

[32] Fabbri E, An Y, Gonzalez-Freire M, Bioavailable Testosterone Linearly Declines Over A Wide Age Spectrum in Men and Women from the Baltimore Longitudinal Study of Aging. The Journals of Gerontology Series A: Biological Sciences and Medical Sciences 71 (2016): 1202–9. 26921861 10.1093/gerona/glw021 PMC4978359

[33] Mulhall JP, Trost LW, Brannigan RE, Evaluation and Management of Testosterone Deficiency: AUA Guideline. Journal of Urology 200 (2018): 423–32. 29601923 10.1016/j.juro.2018.03.115

[34] Bhasin S, Brito JP, Cunningham GR, Testosterone Therapy in Men with Hypogonadism: An Endocrine Society* Clinical Practice Guideline. The Journal of Clinical Endocrinology & Metabolism [Internet] 103 (2018): 1715–44. 29562364 10.1210/jc.2018-00229

[35] Center for Drug Evaluation and Research. FDA Drug Safety Communication: FDA cautions about using testosterone products for low testosterone due to aging; requires labeling change to inform of possible increased risk of heart attack and stroke with use | FDA [Internet]. U.S. Food and Drug Administration (2019). Available from: https://www.fda.gov/drugs/drug-safety-and-availability/fda-drug-safety-communication-fda-cautions-about-using-testosterone-products-low-testosterone-due

[36] Yu J, Akishita M, Eto M, Androgen Receptor-Dependent Activation of Endothelial Nitric Oxide Synthase in Vascular Endothelial Cells: Role of Phosphatidylinositol 3-Kinase/Akt Pathway. Endocrinology 151 (2010): 1822–8. 20194727 10.1210/en.2009-1048

[37] Yu J, Akishita M, Eto M, Src kinase-mediates androgen receptor-dependent non-genomic activation of signaling cascade leading to endothelial nitric oxide synthase. Biochemical and Biophysical Research Communications 424 (2012): 538–43. 22771325 10.1016/j.bbrc.2012.06.151

[38] Puttabyatappa Y, Stallone JN, Adviye Ergul, Peroxynitrite Mediates Testosterone-Induced Vasodilation of Microvascular Resistance Vessels. Journal of Pharmacology and Experimental Therapeutics 345 (2013): 7–14. 23318471 10.1124/jpet.112.201947 PMC3608447

[39] Scragg JL, Jones RD, Channer KS, Testosterone is a potent inhibitor of L-type Ca2+ channels. Biochemical and biophysical research communications 318 (2004): 503–6. 15120629 10.1016/j.bbrc.2004.04.054

[40] Sakamoto K, Kurokawa J. Involvement of sex hormonal regulation of K+ channels in electrophysiological and contractile functions of muscle tissues. Journal of Pharmacological Sciences 139 (2019): 259–65. 30962088 10.1016/j.jphs.2019.02.009

[41] Bai CX, Kurokawa J, Tamagawa M, Nontranscriptional Regulation of Cardiac Repolarization Currents by Testosterone. Circulation 112 (2005): 1701–10. 16157773 10.1161/CIRCULATIONAHA.104.523217

[42] McManus JF, Nguyen NN, Davey RA, Androgens stimulate erythropoiesis through the DNA-binding activity of the androgen receptor in non-hematopoietic cells. European Journal of Haematology 105 (2020): 247–54. 32311143 10.1111/ejh.13431

[43] Guo W, Bachman E, Li M, Testosterone administration inhibits hepcidin transcription and is associated with increased iron incorporation into red blood cells. Aging Cell 12 (2013): 280–91. 23399021 10.1111/acel.12052 PMC3602280

[44] Hennigar SR, Berryman CE, Harris MN, Testosterone Administration During Energy Deficit Suppresses Hepcidin and Increases Iron Availability for Erythropoiesis. The Journal of Clinical Endocrinology & Metabolism [Internet] 105 (2020): e1316–21. 10.1210/clinem/dgz316 31894236

[45] Herbst KL, Amory JK, Brunzell JD, Testosterone administration to men increases hepatic lipase activity and decreases HDL and LDL size in 3 wk. American Journal of Physiology Endocrinology and Metabolism [Internet] 284 (2003): E1112–1118. 12736156 10.1152/ajpendo.00524.2002

[46] Tan KCB, Shiu SWM, Kung AWC. Alterations in hepatic lipase and lipoprotein subfractions with transdermal testosterone replacement therapy. Clinical Endocrinology 51 (1999): 765–9. 10619982 10.1046/j.1365-2265.1999.00882.x

[47] Mohler ER, Ellenberg SS, Lewis CE, The Effect of Testosterone on Cardiovascular Biomarkers in the Testosterone Trials. The Journal of Clinical Endocrinology & Metabolism 103 (2017): 681–8. 10.1210/jc.2017-02243 PMC5800829 29253154

[48] Kilby EL, Kelly DM, Jones TH. Testosterone stimulates cholesterol clearance from human macrophages by activating LXRα. Life Sciences 269 (2021): 119040. 33453241 10.1016/j.lfs.2021.119040

[49] Qiu Y, Yanase T, Hu H, Dihydrotestosterone suppresses foam cell formation and attenuates atherosclerosis development. Endocrinology [Internet] 151 (2010): 3307–16. 20427482 10.1210/en.2009-1268

[50] Høst C, Gormsen LC, Christensen B, Independent Effects of Testosterone on Lipid Oxidation and VLDL-TG Production. Diabetes 62 (2013): 1409–16. 23193189 10.2337/db12-0440 PMC3636625

[51] Malkin CJ, Pugh PJ, Jones RD, The Effect of Testosterone Replacement on Endogenous Inflammatory Cytokines and Lipid Profiles in Hypogonadal Men. The Journal of Clinical Endocrinology & Metabolism 89 (2004): 3313–8. 15240608 10.1210/jc.2003-031069

[52] Keller ET, Chang C, Ershler WB. Inhibition of NFκB Activity through Maintenance of IκBα Levels Contributes to Dihydrotestosterone-mediated Repression of the Interleukin-6 Promoter. J Biol Chem 271 (1996): 26267–75. 8824277 10.1074/jbc.271.42.26267

[53] Ripple MO, Henry WF, Schwarze SR, Effect of Antioxidants on Androgen-Induced AP-1 and NF-B DNA-Binding Activity in Prostate Carcinoma Cells. Journal of the National Cancer Institute 91 (1999): 1227–32. 10413424 10.1093/jnci/91.14.1227

[54] Benten WPM, Lieberherr M, Stamm O, Testosterone Signaling through Internalizable Surface Receptors in Androgen Receptor-free Macrophages. Yamamoto KR, editor. Molecular Biology of the Cell 10 (1999): 3113–23. 10512854 10.1091/mbc.10.10.3113 PMC25566

[55] Estrada M, Espinosa A, Müller M, Testosterone Stimulates Intracellular Calcium Release and Mitogen-Activated Protein Kinases Via a G Protein-Coupled Receptor in Skeletal Muscle Cells. Endocrinology 144 (2003): 3586–97. 12865341 10.1210/en.2002-0164

[56] Pastuszak AW, Gittelman M, Tursi JP, Pharmacokinetics of testosterone therapies in relation to diurnal variation of serum testosterone levels as men age. Andrology 10 (2021): 209–22. 34510812 10.1111/andr.13108 PMC9293229

[57] DailyMed - TESTOSTERONE CYPIONATE injection [Internet]. Nih.gov (2025). Available from: https://dailymed.nlm.nih.gov/dailymed/drugInfo.cfm?setid=78d55bad-4a5b-4e21-aeb8-a4c6346208be

[58] DailyMed - TESTOSTERONE ENANTHATE injection, solution [Internet]. Nih.gov (2025). Available from: https://dailymed.nlm.nih.gov/dailymed/drugInfo.cfm?setid=82a98132-9d5f-40a5-8c4f-f52f2a5de60e

[59] DailyMed - AVEED-testosterone undecanoate injection [Internet]. Nih.gov (2021). Available from: https://dailymed.nlm.nih.gov/dailymed/drugInfo.cfm?setid=f80f025b-17d8-40af-8739-20ce07902045

[60] Nankin HR. Hormone kinetics after intramuscular testosterone cypionate. Fertility and Sterility 47 (1987): 1004–9. 3595893

[61] Yassin AA, Haffejee M. Testosterone depot injection in male hypogonadism: a critical appraisal. Clinical Interventions in Aging [Internet] 2 (2007): 577. 18225458 PMC2686335

[62] Behre H, Abshagen K, Oettel M, Intramuscular injection of testosterone undecanoate for the treatment of male hypogonadism: phase I studies. European Journal of Endocrinology 140 (1999): 414–9. 10229906 10.1530/eje.0.1400414

[63] Schubert M, Minnemann T, Hübler D, Intramuscular Testosterone Undecanoate: Pharmacokinetic Aspects of a Novel Testosterone Formulation during Long-Term Treatment of Men with Hypogonadism. The Journal of Clinical Endocrinology & Metabolism 89 (2004): 5429–34. 15531493 10.1210/jc.2004-0897

[64] DailyMed - TESTOSTERONE gel [Internet]. Nih.gov (2024). Available from: https://dailymed.nlm.nih.gov/dailymed/drugInfo.cfm?setid=68b2375d-0365-4797-bc3d-63531c9f42fd

[65] DailyMed - TESTOSTERONE gel, metered [Internet]. Nih.gov (2025). Available from: https://dailymed.nlm.nih.gov/dailymed/drugInfo.cfm?setid=415057d5-8ed3-42dd-9637-05145c84a9c2

[66] Center for Drug Evaluation and Research Application Number: 021463Orig1s000 LABELING [Internet] (2013) Mar. Available from: https://www.accessdata.fda.gov/drugsatfda_docs/nda/2010/021463Orig1s000Lbl.pdf

[67] Highlights of Prescribing Information - Androderm (testosterone transdermal system) [Internet] (2011). Available from: https://www.accessdata.fda.gov/drugsatfda_docs/label/2011/020489s025lbl.pdf

[68] Mazer N, Bell D, Wu J, Original Research—Endocrinology: Comparison of the Steady-State Pharmacokinetics, Metabolism, and Variability of a Transdermal Testosterone Patch Versus a Transdermal Testosterone Gel in Hypogonadal Men. The Journal of Sexual Medicine 2 (2005): 213–26. 16422889 10.1111/j.1743-6109.2005.20231.x

[69] Swerdloff RS, Wang C, Cunningham G, Long-Term Pharmacokinetics of Transdermal Testosterone Gel in Hypogonadal Men1. The Journal of Clinical Endocrinology & Metabolism 85 (2000): 4500–10. 11134099 10.1210/jcem.85.12.7045

[70] Shoskes JJ, Wilson MK, Spinner ML. Pharmacology of testosterone replacement therapy preparations. Translational Andrology and Urology 5 (2016): 834–43. 28078214 10.21037/tau.2016.07.10 PMC5182226

[71] DailyMed - METHITEST-methyltestosterone tablet [Internet]. Nih.gov (2025). Available from: https://dailymed.nlm.nih.gov/dailymed/drugInfo.cfm?setid=77bb4ef4-c10e-4acc-8225-651d003f4561

[72] DailyMed - METHYLTESTOSTERONE capsule [Internet]. Nih.gov (2025). Available from: https://dailymed.nlm.nih.gov/dailymed/drugInfo.cfm?setid=9bed4886-fcd0-4056-be8d-5bb4d15081ce

[73] DailyMed - TLANDO-testosterone undecanoate capsule, liquid filled [Internet]. Nih.gov (2025). Available from: https://dailymed.nlm.nih.gov/dailymed/drugInfo.cfm?setid=479b55bd-2023-486a-8922-3b1de48b935c

[74] DailyMed - NATESTO NASAL GEL-testosterone gel, metered [Internet]. Nih.gov (2022). Available from: https://dailymed.nlm.nih.gov/dailymed/drugInfo.cfm?setid=dea6bed1-eaca-11e3-ac10-0800200c9a66

[75] DailyMed - STRIANT-testosterone tablet [Internet]. Nih.gov (2025). Available from: https://dailymed.nlm.nih.gov/dailymed/drugInfo.cfm?setid=ac47efbe-025b-4688-bcd3-a10a0b012b34

[76] Wang C, Swerdloff R, Kipnes M, New Testosterone Buccal System (Striant) Delivers Physiological Testosterone Levels: Pharmacokinetics Study in Hypogonadal Men. The Journal of Clinical Endocrinology & Metabolism 89 (2004): 3821–9. 15292312 10.1210/jc.2003-031866

[77] Kim S, Snipes W, Hodgen GD, Pharmacokinetics of a single dose of Buccal testosterone. Contraception 52 (1995): 313–6. 8585889 10.1016/0010-7824(95)00216-w

[78] DailyMed - TESTOPEL-testosterone pellet [Internet]. Nih.gov (2025). Available from: https://dailymed.nlm.nih.gov/dailymed/drugInfo.cfm?setid=a1741a0b-3d4c-42dc-880d-a06e96cce9ef

[79] Handelsman DJ, Conway AJ, Boylan LM. Pharmacokinetics and Pharmacodynamics of Testosterone Pellets in Man. The Journal of Clinical Endocrinology & Metabolism 71 (1990): 216–22. 2115044 10.1210/jcem-71-1-216

[80] Michael Lincoff A, Bhasin S, Flevaris P, Cardiovascular Safety of Testosterone-Replacement Therapy. The New England Journal of Medicine 389 (2023). 10.1056/NEJMc2309389 37733319

[81] Connelly PJ, Owusu Achiaw S, Friday JM, Association Between Long-Term Testosterone Exposure and Major Adverse Cardiovascular Events in Aging Men. Journal of the Endocrine Society [Internet] 9 (2025). 10.1210/jendso/bvaf156 PMC12559020 41163812

[82] Layton JB, Meier CR, Sharpless JL, Comparative Safety of Testosterone Dosage Forms. JAMA Internal Medicine 175 (2015): 1187. 25962056 10.1001/jamainternmed.2015.1573 PMC4494981

[83] Rivero MJ, Ory J, Diaz P, Comparison of Hematocrit Change in Testosterone-deficient Men Treated with Intranasal Testosterone Gel vs Intramuscular Testosterone Cypionate: A Randomized Clinical Trial. The Journal of Urology 210 (2023): 162–70. 37126399 10.1097/JU.0000000000003487

[84] Scala A, Graziani A, Vianello F, Risk of erythrocytosis in transgender individuals undergoing testosterone therapy: a systematic review. Minerva Endocrinology [Internet] 49 (2024). 10.23736/S2724-6507.24.04171-X 39028210

[85] Ory J, Nackeeran S, Balaji NC, Secondary Polycythemia in Men Receiving Testosterone Therapy Increases Risk of Major Adverse Cardiovascular Events and Venous Thromboembolism in the First Year of Therapy. Journal of the American Urological Association 207 (2022): 1295–301. 10.1097/JU.0000000000002437 PMC12721636 35050717

[86] Kohn TP, Agrawal P, Ory J, Rises in Hematocrit Are Associated with an Increased Risk of Major Adverse Cardiovascular Events in Men Starting Testosterone Therapy: A Retrospective Cohort Claims Database Analysis. The Journal of Urology 211 (2023): 285–93. 37948758 10.1097/JU.0000000000003786

[87] Zhang N, Zhang H, Zhang X, The relationship between endogenous testosterone and lipid profile in middle-aged and elderly Chinese men. European Journal of Endocrinology 170 (2014): 487–94. 24394726 10.1530/EJE-13-0802

[88] Mäkinen JI, Perheentupa A, Irjala K, Endogenous testosterone and serum lipids in middle-aged men. Atherosclerosis 197 (2008): 688–93. 17588587 10.1016/j.atherosclerosis.2007.05.009

[89] Harris K, Herati A, Andriole G, 043 Relationship Between Endogenous Testosterone and Lipid Parameters: Insight from REDUCE Study Group Database. The Journal of Sexual Medicine 17 (2020): S22–3.

[90] Kato Y, Shigehara K, Nakashima K, The five-year effects of testosterone replacement therapy on lipid profile and glucose tolerance among hypogonadal men in Japan: a case control study. The Aging Male 23 (2019): 23–8. 30651019 10.1080/13685538.2018.1550060

[91] Clift AK, Huang DR, Auerbach N, Trends in blood-based metabolic and cardiovascular risk profiles in men during treatment for testosterone deficiency: a longitudinal, retrospective cohort study. The Journal of Sexual Medicine [Internet] 22 (2025): 1564–71. 40693884 10.1093/jsxmed/qdaf170

[92] Khazai B, Hill Golden S, Colangelo LA, Association of endogenous testosterone with subclinical atherosclerosis in men: the multi-ethnic study of atherosclerosis. Clinical Endocrinology 84 (2016): 700–7. 26663365 10.1111/cen.12997

[93] Vikan T, Johnsen SH, Schirmer H, Endogenous testosterone and the prospective association with carotid atherosclerosis in men: the Tromsø study. European Journal of Epidemiology 24 (2009): 289–95. 19263227 10.1007/s10654-009-9322-2

[94] Srinath R, Hill Golden S, Carson KA, Endogenous Testosterone and its Relationship to Preclinical and Clinical Measures of Cardiovascular Disease in the Atherosclerosis Risk in Communities Study. The Journal of Clinical Endocrinology & Metabolism 100 (2015): 1602–8. 25584720 10.1210/jc.2014-3934 PMC5393511

[95] Trumble BC, Negrey J, Koebele SV, Testosterone is positively associated with coronary artery calcium in a low cardiovascular disease risk population. Evolution, Medicine and Public Health 11 (2023): 472–84. 38145005 10.1093/emph/eoad039 PMC10746324

[96] Budoff MJ, Ellenberg SS, Lewis CE, Testosterone Treatment and Coronary Artery Plaque Volume in Older Men with Low Testosterone. JAMA 317 (2017): 708. 28241355 10.1001/jama.2016.21043 PMC5465430

[97] Basaria S, Harman SM, Travison TG, Effects of Testosterone Administration for 3 Years on Subclinical Atherosclerosis Progression in Older Men with Low or Low-Normal Testosterone Levels. JAMA 314 (2015): 570. 26262795 10.1001/jama.2015.8881

[98] Yoshihisa A, Suzuki S, Sato Y, Relation of Testosterone Levels to Mortality in Men with Heart Failure. The American Journal of Cardiology 121 (2018): 1321–7. 29580633 10.1016/j.amjcard.2018.01.052

[99] Zhan X, Liu Y, Chen T, The association between serum testosterone level and congestive heart failure in US male adults: data from National Health and Nutrition Examination Survey (NHANES) 2011–2016. Reproductive Biology and Endocrinology 22 (2024). 10.1186/s12958-023-01171-w PMC10759552 38169409

[100] Njoroge JN, Tressel W, Biggs ML, Circulating Androgen Concentrations and Risk of Incident Heart Failure in Older Men: The Cardiovascular Health Study. Journal of the American Heart Association 11 (2022). 10.1161/JAHA.122.026953 PMC9673636 36285783

[101] Yeap BB, Marriott RJ, Antonio L, Associations of Serum Testosterone and Sex Hormone–Binding Globulin With Incident Cardiovascular Events in Middle-Aged to Older Men. Annals of Internal Medicine 175 (2022): 159–70. 34958606 10.7326/M21-0551

[102] Cannarella R, Barbagallo F, Crafa A, Testosterone replacement therapy in hypogonadal male patients with hypogonadism and heart failure: a meta-analysis of randomized controlled studies. Minerva Urology and Nephrology 74 (2021). 10.23736/S2724-6051.21.04307-X 33781026

[103] Tao J, Liu X, Bai W. Testosterone Supplementation in Patients with Chronic Heart Failure: A Meta-Analysis of Randomized Controlled Trials. Frontiers in Endocrinology 11 (2020): 110–0. 32231640 10.3389/fendo.2020.00110 PMC7082858

[104] Navarro-Peñalver M, Perez-Martinez MT, Gómez-Bueno M, Testosterone Replacement Therapy in Deficient Patients with Chronic Heart Failure: A Randomized Double-Blind Controlled Pilot Study. Journal of Cardiovascular Pharmacology and Therapeutics [Internet] 23 (2018): 543–50. 29929385 10.1177/1074248418784020

[105] Theodorakis N, Kreouzi M, Hitas C, Testosterone replacement therapy in heart failure: A systematic review of randomized controlled trials. HORMONES 24 (2025): 679–93. 40234375 10.1007/s42000-025-00658-y

[106] Berger D, Folsom AR, Schreiner PJ, Plasma total testosterone and risk of incident atrial fibrillation: The Atherosclerosis Risk in Communities (ARIC) study. Maturitas 125 (2019): 5–10. 31133217 10.1016/j.maturitas.2019.03.015 PMC6538393

[107] Tran C, Yeap BB, Ball J, Testosterone and the risk of incident atrial fibrillation in older men: further analysis of the ASPREE study. EClinicalMedicine 72 (2024): 102611–1. 38707912 10.1016/j.eclinm.2024.102611 PMC11067494

[108] O’Neal WT, Nazarian S, Alonso A, Sex hormones and the risk of atrial fibrillation: The Multi-Ethnic Study of Atherosclerosis (MESA). Endocrine 58 (2017): 91–6. 28786078 10.1007/s12020-017-1385-3 PMC5693706

[109] Magnani JW, Moser C, Murabito JM, Association of Sex Hormones, Aging, and Atrial Fibrillation in Men. Circulation: Arrhythmia and Electrophysiology 7 (2014): 307–12. 24610804 10.1161/CIRCEP.113.001322 PMC4035016

[110] Xu B, Mo W, Tan X, Associations of Serum Testosterone and Sex Hormone-binding Globulin with Incident Arrhythmias in Men from UK Biobank. The Journal of Clinical Endocrinology & Metabolism [Internet] 109 (2023): e745–56.3 10.1210/clinem/dgad526 37665960

[111] Greenberg DR, Kohn TP, Asanad K, Association of testosterone replacement therapy with atrial fibrillation and acute kidney injury [Internet]. The Journal of Sexual Medicine (2024). 10.1093/jsxmed/qdae138 39487489

[112] Blackwell KM, Buckingham H, Paul KK, Benefits of Testosterone Replacement Therapy in Hypogonadal Males. The Journal of the American Board of Family Medicine 37 (2024): 816–25. 39978846 10.3122/jabfm.2024.240025R1

[113] Bonnet F, Vaduva P, Balkau B, Testosterone therapy and the risk of atrial fibrillation, venous thromboembolism and cardiovascular events in cis men with hypogonadism and trans men. European Journal of Endocrinology [Internet] 193 (2025): 374–82. 40924869 10.1093/ejendo/lvaf183

